# Association of Dietary Fatty Acid Consumption Patterns with Risk of Hyper-LDL Cholesterolemia in Korean Adults

**DOI:** 10.3390/nu12051412

**Published:** 2020-05-14

**Authors:** Eunhee Choi, Seoeun Ahn, Hyojee Joung

**Affiliations:** 1 Department of Public Health Science, Graduate School of Public Health, Seoul National University, Seoul 08826, Korea; tableb4you@snu.ac.kr (E.C.); ase0821@snu.ac.kr (S.A.); 2 Institute of Health and Environment, Seoul National University, Seoul 08826, Korea

**Keywords:** dietary fatty acids, hyper-LDL cholesterolemia, Korean Genome and Epidemiology Study (KoGES), cohort study

## Abstract

This study aimed to identify the association between the risk of hyper-LDL cholesterolemia (hyper-LDLC) and fatty acid consumption patterns (FACPs) using the data from the Korean Genome and Epidemiology Study (KoGES) prospective cohort. A total of 6542 middle-aged Korean adults were included in the analysis. Four FACPs were identified through principal component analysis of the reported intakes of 34 fatty acids (FAs): “long-chain FA pattern”; “short & medium-chain saturated fatty acid (SFA) pattern”; “n-3 polyunsaturated fatty acid (PUFA) pattern”; and “long-chain SFA pattern”. The “long-chain SFA pattern” lowered the risk of hyper-LDLC (relative risk (RR), 0.82; 95% confidence interval (CI), 0.72–0.94; *p* for trend, 0.004) and the “short & medium-chain SFA pattern” increased the risk of hyper-LDLC (RR, 1.17; 95% CI, 1.03–1.32; *p* for trend = 0.004). In sex-stratified analyses, the associations of the “long-chain SFA pattern” (RR, 0.73; 95% CI, 0.58–0.93; *p* for trend = 0.007) and the “short & medium-chain SFA pattern” (RR, 1.34; 95% CI, 1.07–1.69; *p* for trend = 0.003) with the hyper-LDLC risk were observed only in men, but not in women. These results suggest that FACPs with a high intake of long-chain SFA or a low intake of short and medium-chain SFA may protect Korean adults from hyper-LDLC.

## 1. Introduction

Cardiovascular diseases (CVD) are the leading cause of death worldwide, accounting for 17.9 million deaths in 2016, which is 31% of all global deaths [1]. The mortality rate due to CVD in Korea was 60.2 per 100,000 persons, representing the second most common cause of death after cancer in 2017 [2]. Coronary heart disease (CHD), which is a disease of the blood vessels supplying blood to the heart muscle, accounts for about 46% of deaths from CVD in Korea [2].

Increased plasma levels of low-density lipoprotein cholesterol (LDL-C) have been shown to increase the risk of CHD [3]. The National Cholesterol Education Program Adult Treatment Panel III (NCEP ATP III) identified that an elevated LDL-C is the primary intervention target of cholesterol-lowering therapy [4]. For the management of blood LDL-C levels, lifestyle behaviors such as diet, physical activity, and smoking are major modifiable factors [5,6]. Regarding diet, low total fat intake has been generally recommended for the management of dyslipidemia [5]. Over the decades, dietary guidelines regarding fat intake have mainly aimed at reducing total fat or saturated fatty acid (SFA) intake. However, recently fat quality has come into focus [7], and recommendations for dietary fatty acids (FAs) have been issued in many countries [8,9,10,11,12]. In other words, it is important to consider the intake of the subtypes of FAs as well as total fat intake.

Dietary FAs have different effects on blood cholesterol levels depending on their carbon chain length, the number of double bonds, and double-bond positions [13]. Several epidemiological studies have reported that subtypes of FAs have different impacts on blood cholesterol [14]. For examples, some studies reported that palmitic acid (C16:0) and lauric acid (C12:0) had an LDL-raising effect [15,16], whereas stearic acid (C18:0) had an LDL-lowering effect [15]. In addition, a diet high in stearic acid (C18:0), linoleic acid (C18:2), and α-linolenic acid (C18:3, n-3) significantly lowered LDL-C [16,17].

Recently, the association of dietary fat intake with health outcomes, considering its quality, has been reported. Santiago et al., have shown no effects of dietary fat subtypes on cardiovascular events in the adult population of Spain using a dietary fat quality index, which was calculated according to the ratio (monounsaturated fatty acid (MUFA) + polyunsaturated fatty acid (PUFA))/(SFA + total fatty acid (TFA)) [18]. Noel et al., have examined the association between metabolic syndrome and the combination of FAs intake using principal component analysis, and reported that the pattern with a high intake of n-3 PUFA was associated with a lower likelihood of having metabolic syndrome in the adult population of the US [19]. To date, no previous study has examined the association between hyper-LDL cholesterolemia (hyper-LDLC) and fatty acid consumption patterns (FACPs).

On the other hand, previous studies have shown that sex hormones affect LDL-C levels differently in men and women due to the drop in estrogen during menopause, which leads to an increase in LDL-C [20,21,22]. According to the Korean Society of Lipid and Atherosclerosis, the prevalence of hyper-LDLC among Korean women aged 50 to 59 years was 26.0%, whereas Korean men in that ages showed 18.4% [23]. Therefore, further research on sex differences in associations of FACPs with hyper-LDLC among Korean adults is necessary.

This study aimed to identify the association between the risk of hyper-LDLC and FACPs using the large prospective cohort data from the Korean Genome and Epidemiology Study (KoGES). In addition, we evaluated sex differences in associations between FACPs and hyper-LDLC risk.

## 2. Materials and Methods

### 2.1. Study Design and Subjects

The Korean Genome and Epidemiology Study (KoGES) was designed to investigate risk factors for chronic diseases in a population-based sample of Korean adults. The baseline examinations of the Ansan (urban) and Ansung (rural) study as a part of KoGES were conducted in 2001–2002 for a total of 10,030 adults aged 40–69 years, and the participants were followed up biennially through 2015–2016. Of the 10,030 participants in the cohort, we excluded those who did not participate in a food frequency questionnaire (FFQ) survey (*n* = 326), reported implausible energy intake (< 500 kcal/day or > 5000 kcal/day) (*n* = 85), had chronic diseases such as diabetes, hypertension, any cancer or CVD (myocardial infarction, coronary artery disease, cerebrovascular disease, or congestive heart failure) (*n* = 2529), or had hyper-LDLC (*n* = 548) at baseline. Finally, a total of 6542 subjects were included in the analysis. This study was approved by the Institutional Review Board of the Korea Centers for Disease Control and Prevention and Seoul National University (IRB No. E1910/003–001). Informed consent was obtained from all participants.

### 2.2. Assessment of Dietary Intake

Dietary intake was assessed with the semi-quantitative FFQ including 103 food items conducted at the baseline examination in 2001–2002. The validity and reproducibility of the FFQ were evaluated in the previous study in detail [24]. Participants responded to the frequency and portion size of each food item over the past year. Daily food and nutrient intakes were estimated using the food composition table of the Korean Nutrition Society [25]. We estimated the participants’ daily intakes of 34 FAs by combining FFQ data with the food composition tables from the Rural Development Administration and the Rural Nutrition Institute [26], Korea Institute for Health and Social Affairs [27], Standard Tables of Food Composition in Japan 2015 (7th revised edition) [28], and the USDA Food Composition Database [29]. This developed FAs database covered 98.7% of all ingredients in the listed foods and dishes of the FFQ (391 of 395 ingredients). The lists of 34 FAs, composed of 16 SFA, 7 MUFA, and 11 PUFA, are shown in Table 1. 

### 2.3. Identification of Dietary Fatty Acid Consumption Pattern

A principal component analysis was performed to derive dietary FACPs. An orthogonal rotation (the varimax option in SAS) was used to derive independent dietary FACP to achieve a simpler structure with greater interpretability. For deriving FACPs, the percentages of energy from the 34 FAs were used as input variables to adjust for individual’s daily energy intake. Finally, four significant FACPs were identified considering eigenvalue (> 1), scree plot, and interpretable possibility [30]. Participants were grouped into tertiles (T) according to the factor score of each pattern. 

### 2.4. Ascertainment of Hyper-LDL Cholesterolemia

Fasting blood samples of participants were collected at baseline and every two years during the follow-up period by the trained staffs. Serum levels of total cholesterol, HDL-cholesterol, and triglycerides were measured using Bayer Reagent Packs (Bayer Diagnostics, Leverkusen, Germany) on an automated chemistry analyzer (Adiva 1650 Autoanalyzer; Bayer Diagnostics, Leverkusen, Germany) [31]. The LDL-C levels were calculated using the Friedewald formula [32], and LDL-C levels ≥ 160 mg/dL were defined as hyper-LDLC based on the criteria of the Committee for the Korean Guidelines for the Management of Dyslipidemia [5].

### 2.5. Measurement of Covariates

All socio-demographic information was collected through a self-administered questionnaire regarding sex, age, residential area, smoking status, alcohol consumption status, regular supplementation use, household income level, occupation, regular physical activity, and chronic diseases at baseline. The age was categorized in the 5-year interval, and status of smoking and alcohol consumption were categorized as “never, past, or current”. Regular supplementation use was categorized as “yes or no”. The household income level was divided into “< 1,000,000, 1-<2,000,000, 2-<4,000,000, or ≥ 4,000,000 Korean won (KRW) per month”. The occupation was classified into “office workers, non-office workers, or house workers and others”. Physical activity was estimated using the metabolic equivalents (METs)-hours per day. For quantitative estimation of physical activity, a weighted value was allocated to each activity considering their intensity, and the weighted value of each activity was multiplied by the time of that activity [33]. Then, all these values summed up to the total MET for each person.

Anthropometric variables such as body weight and height were measured by the trained examiners. Body mass index (BMI) was calculated by dividing the weight (kg) by the height squared (m^2^) and then categorized into “underweight (< 18.5 kg/m^2^), normal weight (18.5-<23 kg/m^2^), overweight (23-<25 kg/m^2^), or obese (≥ 25 kg/m^2^)” status, based on the WHO obesity criteria for the Asian-Pacific region [34].

### 2.6. Statistical Analysis

The baseline general characteristics of the participants are shown as mean ± standard deviation for the continuous variables or *n* (%) for categorical variables in each tertile of the FACP score. The differences of general characteristics according to the tertiles of each FACP score were compared using the ANOVA test for continuous variables and the chi-square test for categorical variables. Using the Spearman correlation analysis, the associations between each pattern score with the intakes of 18 food groups, energy, and nutrients were assessed. The person-time of each participant was calculated from the enrollment date to the most recent follow-up survey date in case of a person without hyper-LDLC and to incident date of hyper-LDLC in case of a person with hyper-LDLC. Cox proportional hazard regression analysis was carried out to estimate the relative risks (RR) and 95% confidence intervals (95% CI). The lowest score groups of each FACP were used as the reference. All multivariable-adjusted models were adjusted for age, sex (except for sex-stratified models), residence, smoking status, alcohol consumption status, regular supplementation use, physical activity, BMI, household income level, occupation, and daily energy intake. The *p*-value for interaction was obtained via the likelihood ratio test using models with and without the interaction terms (sex × tertiles of FACP score). All statistical analyses were performed using the SAS software version 9.4 (SAS Institute, Cary, NC, USA). All *p*-values were two-sided, and a *p*-value < 0.05 was considered statistically significant.

## 3. Results

### 3.1. Dietary Fatty Acid Consumption Pattern (FACP)

Principal component analysis of 34 individual FAs intakes revealed four significant FACP factors, accounting for 26.81% of the variance. Pattern 1 (Long-chain FA pattern), pattern 2 (Short and medium-chain SFA pattern), pattern 3 (n-3 PUFA pattern), and pattern 4 (Long-chain SFA pattern) explained 8.16%, 7.72%, 6.90%, and 4.03% of FAs intake variance, respectively. The factor loading matrix of FAs is shown in Table 2.

The “long-chain FA pattern” was characterized by high positive loadings of long-chain PUFA such as C20:3, n-3 and C20:2, n-6, long-chain SFA such as C18:0 and C16:0, and long-chain MUFA such as C18:1 and C16:1. The “short & medium-chain SFA pattern” was characterized by high loadings of short and medium-chain SFA, such as C6:0, C10:0, and C4:0. The “n-3 PUFA pattern” was characterized by high loadings of n-3 PUFA such as C20:5, n-3 (EPA), C22:6, n-3 (DHA), and C22:5, n-3 (DPA). The “long-chain SFA pattern” showed high loadings of long-chain SFA, such as C22:0, C24:0, and C20:0.

The correlations of the four FACP scores with intakes of nutrients and food groups are shown in Table 3. The “long-chain FA pattern” represented a diet relatively high in MUFA, fat, SFA, protein, PUFA, and n-6 PUFA (*r* = 0.84, 0.79, 0.74, 0.53, 0.46 and 0.46, respectively, all *p* < 0.0001), but low in carbohydrates (*r* = −0.75, *p* < 0.0001), and its score was positively correlated to the intakes of “meat and meat products” and “poultry” groups (*r* = 0.87 and 0.40, respectively, all *p* < 0.0001). The “short & medium-chain SFA pattern” represented a diet relatively high in SFA (*r* = 0.57, *p* < 0.0001), and its score was positively correlated to the intakes of the “milk and dairy” and “snack” groups (*r* = 0.79 and 0.35, all *p* < 0.0001). The “n-3 PUFA pattern” represented a diet relatively high in n-3 PUFA and protein (*r* = 0.66 and 0.52, respectively, all *p* < 0.0001), and showed a positive association with the intakes of the “fish and shellfish” and the “seaweeds” groups (*r* = 0.76 and 0.33, respectively, all *p* < 0.0001). The “long-chain SFA pattern” represented a diet relatively high in n-6 PUFA, PUFA, and n-3 PUFA (*r* = 0.84, 0.82 and 0.58, respectively, all *p* < 0.0001), and its score was positively associated with the “bean and tofu” and “nuts and seeds” groups (*r* = 0.54 and 0.40, respectively, all *p* < 0.0001).

### 3.2. General Characteristics of Study Subjects

Table 4 shows the general characteristics of the study subjects, according to the tertiles (T) of each FACP score. Participants in the highest tertile of the “long-chain FA pattern” score had a significantly higher proportion of men (*p* < 0.0001), whereas that of the “short & medium-chain SFA pattern” had a significantly lower proportion of men (*p* < 0.0001). Participants in the highest tertile of the “long-chain FA pattern” and “n-3 PUFA pattern” score were more likely to drink alcohol (*p* < 0.0001 and *p =* 0.0009, respectively), but those of the “short & medium-chain SFA pattern” score were less likely to drink alcohol (*p =* 0.0093). Participants in the highest tertile of the “long-chain FA pattern” score were more likely to be current smokers (*p* < 0.0001), but those of the “short & medium-chain SFA pattern” score tended to smoke less (*p =* 0.0010). Participants in the highest tertile of the “n-3 PUFA pattern” score had a higher BMI (*p* < 0.0001). Those in the highest tertile of the “short & medium-chain SFA pattern”, “n-3 PUFA pattern”, and “long-chain SFA pattern” score tended to use more supplementation (*p* < 0.0001, *p =* 0.0015, and *p* < 0.0001, respectively). As for the energy and nutrient intakes, a higher FACP score was positively correlated with the intakes of energy and all nutrients except carbohydrate in the “long-chain FA pattern”, “n-3 PUFA pattern”, and long-chain SFA pattern” (all *p* < 0.0001). In the “short & medium-chain SFA pattern”, a higher FACP score was positively correlated with the intake of energy and all nutrients except PUFA, n-3 PUFA, and n-6 PUFA intakes (all *p* < 0.0001). In all patterns, a higher FACP score was negatively correlated with carbohydrate intake (all *p* < 0.0001).

### 3.3. Association between Fatty Acid Consumption Patterns and Hyper-LDL Cholesterolemia

During a median follow-up of 12.5 person-years (interquartile range, 3.5 to 13.8), a total of 1502 cases of hyper-LDLC (23.0%) were identified among 6542 participants without hyper-LDLC at baseline. Table 5 presents the RR and 95% CI for hyper-LDLC across the tertile categories for the four FACP scores. An inverse association between the “long-chain SFA pattern” score and the incidence of hyper-LDLC was observed. Among the total population, those in the highest tertile of the “long-chain SFA pattern” score had an 18% decreased risk of hyper-LDLC compared with the lowest tertile, with a significant linear trend (RR, 0.82; 95% CI, 0.72–0.94; *p* for trend, 0.004). However, the risk of hyper-LDLC in the highest tertile of the “short & medium-chain SFA pattern” score, compared to the lowest tertile, increased by 17% in the multivariate-adjusted model with a significant linear trend (RR, 1.17; 95% CI, 1.03–1.32; *p* for trend = 0.004). The “long-chain FA pattern” and “n-3 PUFA pattern” were not associated with hyper-LDLC.

We examined whether the effects of FACPs on the risk of hyper-LDLC differ between sexes. Among men but not women, the risk of hyper-LDLC was increased significantly in those at the highest tertile of the “short & medium-chain SFA pattern” score (RR, 1.34; 95% CI, 1.07–1.69; *p* for trend = 0.003; *p* for interaction = 0.042), whereas the risk of hyper-LDLC was decreased significantly in those at the highest tertile of the “long-chain SFA pattern” score (RR, 0.73; 95% CI, 0.58–0.93; *p* for trend = 0.007; *p* for interaction = 0.070). The associations of the “long-chain FA pattern” and “n-3 PUFA pattern” with hyper-LDLC risk were not significantly different between men and women.

## 4. Discussion

In this study of a middle-aged Korean population, four major FACPs—the “long-chain FA pattern”, “short & medium-chain SFA pattern”, “n-3 PUFA pattern”, and “long-chain SFA pattern”—were identified. The “short & medium-chain SFA pattern” was positively associated with hyper-LDLC risk, whereas the “long-chain SFA pattern” was associated with decreased hyper-LDLC risk. 

To our knowledge, there has been no attempt to assess the effect of dietary FACPs on hyper-LDLC risk, but the major FACPs identified in this study, the “short & medium-chain SFA pattern”, “n-3 PUFA pattern”, and “long-chain SFA pattern”, were very similar to those observed among Puerto Rican adults living in the US [18]. In the Puerto Rican population, 4 FACPs—the “SFA pattern”, “n-3 PUFA pattern”, “very-long-chain SFA & PUFA pattern”, and “MUFA & TFA pattern”—were identified. The “SFA pattern”—which was characterized by high loadings of capric acid (C10:0), myristic acid (14:0), lauric acid (C12:0), and caproic acids (C6:0)—was inversely associated with fasting serum glucose concentration and the “very-long-chain SFA & PUFA pattern”—which was characterized mainly by linolenic acid (C18:3) and long-chain SFA such as arachidic acid (C20:0) and behenic acid (C22:0)—was negatively related to waist circumference. In spite of the different outcome variables, the identified FACPs were very similar to those found in our analysis. 

The “long-chain SFA pattern” in this study—which was characterized by a high intake of long-chain SFA such as behenic acid (C22:0), lignoceric acid (C24:0), and arachidic acid (C20:0), and n-6 PUFA, linoleic acid (C18:2)—was inversely associated with the risk of hyper-LDLC. To date, very limited studies regarding the effects of dietary long-chain SFA with carbon chain lengths more than 20 on blood lipid levels have been conducted, and only dietary behenic acid (C22:0) was found to increase LDL-C levels [35]. However, their bioavailability is low compared to other FAs due to their long-chain length [35,36]. In a case-control study with adults aged 25–74 years in the US, higher levels of erythrocyte membrane SFA such as behenic acid (C22:0) and lignoceric acid (C24:0) were associated with lower risk of sudden cardiac arrest [37]. In the large prospective cohort studies in the US, inverse associations of plasma long-chain SFA with coronary heart diseases and atrial fibrillation were observed [38,39]. However, there is a lack of evidence regarding the effect of dietary long-chain SFA with carbon chain lengths more than 20 on blood lipid profiles. Therefore, further research is needed for better understanding of the effect of dietary long-chain SFA on blood LDL-C level. In addition, linoleic acid (C18:2, n-6) also contributed highly to this pattern. According to previous studies, there is a significant body of evidence to support the LDL-C lowering effect of linoleic acid (C18:2, n-6) [40,41,42,43]. Considering that people consume FAs combinations from various food sources in general, the results imply that the dietary pattern with a high intake of a mixture of long-chain SFA and n-6 PUFA have beneficial effects on lowering LDL-C level.

However, those adhering more to the “short & medium-chain SFA pattern” were found to be at a higher risk of hyper-LDLC. The “short & medium-chain SFA pattern” had a high factor loading of medium-chain SFA, capric acid (C10:0), and short-chain SFA such as caproic acid (C6:0) and butyric acid (C4:0). Short and medium-chain SFAs are major sources of energy [44], attenuate weight gain [45], and have strong antibacterial effects [46], but there is a lack of evidence for the effect of short-chain SFA on blood LDL-C. Meanwhile, previous studies have reported that the medium-chain SFA would increase serum cholesterol concentration due to stimulation of insulin secretion and de novo FA synthesis, which leads to very-low-density lipoprotein secretion into the bloodstream [47,48,49,50,51]. The participants who strongly adhered to the “short & medium-chain SFA pattern” in this study tended to consume the short and medium-chain SFA from not only milk, but also milk-based confectionery, such as coffee creamer, ice cream, and bakery products, which contain a high level of sugar that is known to have a negative impact on cardiovascular health [52,53,54,55].

Our sex stratified analyses indicated that the effects of the “long-chain SFA pattern” and “short & medium-chain SFA pattern” on hyper-LDLC risk differed between sexes. In order to understand why the association between the “long-chain SFA pattern” and the hyper-LDLC risk appeared only in men but not in women, it is important to compare the general characteristics and dietary intakes between men and women. At baseline, men in the highest tertile of the “long-chain SFA pattern” score had a higher intake of n-6 PUFA, which has an LDL-C lowering effect, than women (men, 3.4 ± 0.7 percentage of energy (%E); women, 3.3 ± 0.7 %E; *p <* 0.0001). In addition, men in the highest tertile of this pattern score were more physically active than women, which would reinforce the LDL-C lowering effect of the n-6 PUFA in men (men, 23.1 ± 14.9 METs-hours per day; women 21.7 ± 13.9 METs-hours/day; *p =* 0.031) [56].

The “short & medium-chain SFA pattern” was associated with an increased risk of hyper-LDLC in men, but not in women. We compared the major dietary food sources contributing to this pattern to identify the sex differences. In women with higher pattern score of “short & medium-chain SFA pattern”, milk and yogurt intake were significantly higher than men (all *p <* 0.0001), but coffee creamer and pork belly intake were significantly lower than men (all *p <* 0.0001). Thus, one possible explanation for this finding might be that short and medium-chain SFA intake through high-sugar and high-fat foods, such as coffee creamer and pork belly, may strengthen an LDL-C raising effect of “short and medium-chain SFA pattern” among Korean men.

The “long-chain FA pattern” and “n-3 PUFA pattern” were not associated with hyper-LDLC in this study. The “long-chain FA pattern” was characterized by a high intake of long-chain PUFA such as icosatrienoic acid (C20:3, n-3) and eicosadienoic acid (C20:2, n-6), and long-chain SFA such as stearic acid (C18:0). In previous studies, stearic acid (C18:0), as compared with other SFA, lowers LDL-C level slightly [57,58,59,60,61], but the main sources of long-chain FAs in our population was meat, that was positively associated with LDL-C level due to its high content of total SFA and cholesterol [62,63,64,65]. Because of the coexistence of factors with opposite effects on LDL-C level, our study could not confirm the effect of long-chain FAs on LDL-C level, so further studies are required to illuminate the effects of long-chain FAs on blood lipid profiles depending on the food sources. The “n-3 PUFA pattern” identified in this study was characterized by a high intake of n-3 PUFA, such as EPA (C20:5, n-3) and DHA (C22:6, n-3), and long-chain MUFA such as nervonic acid (C24:1) and erucic acid (C22:1). The intake of n-3 PUFA in all tertiles of the “n-3 PUFA pattern” score was lower than the recommended intake for Korean, so the actual effect of n-3 PUFA on blood lipids could be alleviated by low intake of n-3 PUFA, although several systematic review and meta-analysis reported that EPA and DHA showed to increase LDL-C level slightly [66,67,68].

To the best of our knowledge, this is the first study to reveal a causal relationship between dietary FACPs and the risk of hyper-LDLC in Korean adults using data from a large-scale cohort study. In real life, people eat mixed diets consisting of various FAs, not isolated one FA. Thus, the FAs pattern analysis considering the subtypes of FAs is important because it can reflect actual diet quality with summarizing effects of various dietary FAs. However, this study has several limitations. First of all, intakes of trans fatty acids and FAs from supplements were not included in the analysis, therefore, the intake of certain FAs was likely to be underestimated. Thus, an improved FAs database for common Korean foods and supplements is required to elucidate the association between comprehensive FAs intake and blood lipid profiles. Second, data for the dietary FAs consumption and socio-demographic information only at baseline were used, so it may not represent long-term status during the follow-up period. The results in this study should be interpreted with caution, owing to the possibility of dietary changes during the follow-up period. Therefore, further research considering dietary changes is needed to apply the results to the general population. Third, this study—with subjects from only two specific areas—could not represent the characteristics of whole Korean population, but could infer the situations of the rural and urban areas in Korea. Lastly, although we adjusted for potential confounders, there may be unrevealed confounding factors that could have an effect on the risk of hyper-LDLC. Notwithstanding these limitations, to our knowledge, this is the first study to prospectively examine the effects of FACPs on the risk of hyper-LDLC.

## 5. Conclusions

In summary, we found that the “long-chain SFA pattern” was inversely associated with the incidence of hyper-LDLC, and the “short & medium-chain SFA pattern” was positively associated with hyper-LDLC in men. The results of this large prospective cohort study suggest that FACPs with a high intake of long-chain SFA and n-6 PUFA or a low intake of short and medium-chain SFA may protect against hyper-LDLC, and provide evidences for developing dietary recommendations for cardiometabolic health, taking dietary FACPs into account.

## Figures and Tables

**Table 1:** The lists of 34 subtypes of fatty acids included in the fatty acid database.

Saturated Fatty Acids (SFA)	Monounsaturated Fatty Acids (MUFA)	Polyunsaturated Fatty Acids (PUFA)
Butyric acid (C4:0)	Myristoleic acid (C14:1)	Linoleic acid (C18:2, n-6)
Caproic acid (C6:0)	Palmitoleic acid (C16:1)	α-linolenic acid (C18:3, n-3)
Caprylic acid (C8:0)	Heptadecenoic acid (C17:1)	γ-linolenic acid (C18:3, n-6)
Capric acid (C10:0)	Oleic acid (C18:1)	Eicosadienoic acid (C20:2, n-6)
Lauric acid (C12:0)	Gadoleic acid (C20:1)	Icosatrienoic acid (C20:3, n-3)
Tridecanoic acid (C13:0)	Erucic acid (C22:1)	Eicosatrienoic acid (C20:3, n-6)
Myristic acid (C14:0)	Nervonic acid (C24:1)	Arachidonic acid (C20:4, n-6)
Pentadecanoic acid (C15:0)		Eicosapentaenoic acid (EPA)(C20:5, n-3)
Palmitic acid (C16:0)		Docosadienoic acid (C22:2)
Heptadecanoic acid (C17:0)		Docosapentaenoic acid (DPA)(C22:5, n-3)
Stearic acid (C18:0)		Docosahexaenoic acid (DHA)(C22:6, n-3)
Arachidic acid (C20:0)		
Henicosanoic acid (C21:0)		
Behenic acid (C22:0)		
Tricosanoic acid (C23:0)		
Lignoceric acid (C24:0)		

**Table 2:** Factor loading matrix of FAs for the major FACPs ^1.^

	Pattern 1	Pattern 2	Pattern 3	Pattern 4
	Long-chain FA pattern	Short and medium-chain SFA pattern	n-3 PUFA pattern	Long-chain SFA pattern
PUFA				
C18:2, n-6	0.42	-	-	0.83
C18:3, n-3	-	-	-	0.74
C18:3, n-6	-	-	0.69	-
C20:2, n-6	0.95	-	-	-
C20:3, n-3	0.96	-	-	-
C20:3, n-6	0.79	0.48	-	-
C20:4, n-6	0.72	-	0.40	-
C20:5, n-3	-	-	0.94	-
C22: 2	-	-	0.41	-
C22:5, n-3	0.34	-	0.88	-
C22:6, n-3	-	-	0.93	-
SFA				
C4:0	-	0.92	-	-
C6:0	-	0.95	-	-
C8:0	-	0.68	-	-
C10:0	-	0.93	-	-
C12:0	-	0.60	-	-
C13:0	-	0.90	-	-
C14:0	0.35	0.89	-	-
C15:0	-	0.87	0.34	-
C16:0	0.80	0.39	-	-
C17:0	0.75	0.49	0.35	-
C18:0	0.90	0.37	-	-
C20:0	0.51	0.30	-	0.69
C21:0	-	0.52	-	0.43
C22:0	-	-	-	0.91
C23:0	0.34	-	-	-
C24:0	-	-	-	0.88
MUFA				
C14:1	0.32	0.70	-	-
C16:1	0.81	-	0.49	-
C17:1	-	-	0.77	-
C18:1	0.86	-	-	0.36
C20:1	0.75	-	0.57	-
C22:1	-	-	0.85	-
C24:1	-	-	0.93	-
Variability (%) ^2^	8.16	7.72	6.90	4.03

^1^ Absolute values less than 0.30 are not listed for clarity. ^2^ Variance of intake explained.

**Table 3:** Spearman correlation coefficients between FACP score and intakes of energy, macronutrients, and food groups in study subjects.

	Pattern 1: Long-Chain FA Pattern		Pattern 2: Short and Medium-Chain SFA Pattern		Pattern 3: n-3 PUFA Pattern		Pattern 4: Long-Chain SFA Pattern	
	Item	r ^1^	Item	r	Item	r	Item	r
Energy and Macronutrients ^2^	Energy	0.23	Energy	0.13	Energy	0.12	Energy	0.24
	Carbohydrates	−0.75	Carbohydrates	−0.31	Carbohydrates	−0.36	Carbohydrates	−0.45
	Fat	0.79	Fat	0.36	Fat	0.26	Fat	0.43
	SFA	0.74	SFA	0.57	SFA	0.19	SFA	0.28
	MUFA	0.84	MUFA	0.23	MUFA	0.28	MUFA	0.39
	PUFA	0.46	PUFA	0.02	PUFA	0.20	PUFA	0.82
	n-3 PUFA	0.32	n-3 PUFA	0.04	n-3 PUFA	0.66	n-3 PUFA	0.58
	n-6 PUFA	0.46	n-6 PUFA	0.01	n-6 PUFA	0.07	n-6 PUFA	0.84
	Protein	0.53	Protein	0.14	Protein	0.52	Protein	0.43
Food groups ^3^	Meat and meat products	0.87	Milk and dairy products	0.79	Fish and shellfish	0.76	Bean and tofu	0.54
	Poultry	0.40	Snack	0.35	Seaweeds	0.33	Nuts and seeds	0.40
	Fish and shellfish	0.31			Mushroom	0.33	Noodles and bread	0.39

^1^ Spearman’s correlation coefficients; ^2^ All correlation coefficients are significant, all *p* < 0.0001, except for PUFA (*p =* 0.177), n-3 PUFA (*p =* 0.001), and n-6 PUFA *(p =* 0.318) in pattern 2; ^3^ Absolute values more than 0.30 are listed, and all listed correlations are significant, all *p* < 0.0001.

**Table 4:** Baseline characteristics of the study subjects according to the tertiles (T) of the FACP score.

	Long-Chain FA Pattern		*p* -Value ^1^	Short and Medium-Chain SFA Pattern		*p* -Value	n-3 PUFA Pattern		*p* -Value	Long-Chain SFA Pattern		*p* -Value
	T1 (lowest)	T3 (highest)		T1	T3		T1	T3		T1	T3	
*N*	2180	2181		2180	2181		2180	2181		2180	2181	
Age (years)	53.4 ± 9.1 ^2^	48.6 ± 7.7	<0.0001	52.7 ± 9.0	49.5 ± 8.2	<0.0001	53.3 ± 9.1	48.8 ± 7.8	<0.0001	52.2 ± 8.8	49.5 ± 8.4	<0.0001
Men (%)	750 (34.4)	1284 (58.9)	<0.0001	1018 (46.7)	962 (44.1)	<0.0001	1082 (49.6)	987 (45.3)	0.0147	1024 (47.0)	1033 (47.4)	0.6541
Urban residence ^3^	896 (41.1)	1319 (60.5)	<0.0001	785 (36.0)	1401 (64.2)	<0.0001	762 (35.0)	1477 (67.7)	<0.0001	870 (40.0)	1338 (61.4)	<0.0001
Higher income ^4^	99 (4.5)	195 (8.9)	<0.0001	106 (4.9)	202 (9.3)	<0.0001	88 (4.0)	226 (10.4)	<0.0001	82 (3.8)	226 (10.4)	<0.0001
Office workers	107 (4.9)	249 (11.4)	<0.0001	129 (5.9)	230 (10.6)	<0.0001	141 (6.5)	234 (10.7)	<0.0001	160 (7.3)	222 (10.2)	<0.0001
Physical activity ^5^	24.5 ± 15.8	22.8 ± 15.0	<0.0001	25.4 ± 16.3	22.0 ± 14.1	<0.0001	26.0 ± 16.3	20.9 ± 13.7	<0.0001	24.2 ± 15.9	22.4 ± 14.6	0.0009
Current alcohol use	804 (36.9)	1321 (60.6)	<0.0001	1078 (49.5)	1038 (47.6)	0.0093	1006 (46.2)	1117 (51.2)	0.0009	1046 (48.0)	1122 (51.4)	0.2406
Current smoking	424 (19.5)	719 (33.0)	<0.0001	584 (26.8)	539 (24.7)	0.0010	623 (28.6)	557 (25.5)	0.0689	603 (27.7)	554 (25.4)	0.1127
BMI ^6^	24.2 ± 3.3	24.2 ± 3.0	0.6558	24.3 ± 3.1	24.1 ± 3.0	0.1402	23.9 ± 3.1	24.5 ± 3.0	<0.0001	24.1 ± 3.1	24.2 ± 3.1	0.0954
Supplementation ^7^	402 (18.4)	408 (18.7)	0.9704	342 (15.7)	487 (22.3)	<0.0001	362 (16.6)	454 (20.8)	0.0015	342 (15.7)	469 (21.5)	<0.0001
Nutrient intake												
Total energy ^8^	1824.6 ± 616.6	2123.7 ± 648.2	<0.0001	1854.3 ± 627.1	2010.7 ± 609.5	<0.0001	1891.1 ± 626.1	2039.1 ± 644.8	<0.0001	1812.5 ± 613.9	2077.9 ± 633.5	<0.0001
Carbohydrates ^9^	76.3 ± 4.9	64.7 ± 5.9	<0.0001	73.1 ± 7.2	68.4 ± 6.2	<0.0001	73.2 ± 6.9	67.5 ± 6.6	<0.0001	74.1 ± 7.3	67.5 ± 6.0	<0.0001
Protein ^9^	12.2 ± 2.0	14.8 ± 2.2	<0.0001	13.1 ± 2.5	13.8 ± 2.1	<0.0001	12.2 ± 1.9	14.9 ± 2.3	<0.0001	12.4 ± 2.4	14.4 ± 2.1	<0.0001
Fat ^9^	11.6 ± 3.3	20.5 ± 4.3	<0.0001	13.8 ± 5.2	17.8 ± 4.6	<0.0001	14.6 ± 5.4	17.6 ± 4.9	<0.0001	13.6 ± 5.3	18.1 ± 4.6	<0.0001
SFA ^9^	3.5 ± 1.6	7.2 ± 1.9	<0.0001	3.9 ± 2.0	6.7 ± 1.9	<0.0001	4.9 ± 2.4	5.8 ± 2.1	<0.0001	4.6 ± 2.4	5.9 ± 2.1	<0.0001
MUFA ^9^	2.4 ± 1.1	6.1 ± 1.8	<0.0001	3.7 ± 2.2	4.6 ± 1.8	<0.0001	3.6 ± 2.1	4.8 ± 1.9	<0.0001	3.4 ± 2.1	4.9 ± 1.9	<0.0001
PUFA ^9^	2.7 ± 0.9	3.7 ± 0.8	<0.0001	3.1 ± 1.0	3.2 ± 0.9	0.1656	3.0 ± 1.0	3.4 ± 0.9	<0.0001	2.4 ± 0.6	4.0 ± 0.8	<0.0001
n-3 PUFA ^9^	0.4 ± 0.2	0.6 ± 0.2	<0.0001	0.5 ± 0.2	0.5 ± 0.2	0.0911	0.4 ± 0.1	0.7 ± 0.2	<0.0001	0.4 ± 0.2	0.6 ± 0.2	<0.0001
n-6 PUFA ^9^	2.3 ± 0.8	3.1 ± 0.7	<0.0001	2.6 ± 0.9	2.7 ± 0.7	0.3842	2.6 ± 0.9	2.7 ± 0.7	<0.0001	2.0 ± 0.5	3.4 ± 0.7	<0.0001

^1^ *p*-value based on ANOVA test for continuous variables (mean ± SD), and $\chi^{2}$ test for categorical variables (*n*, (%)). ^2^ mean ± SD (all such values). ^3^ urban or rural. ^4^ Household income ≥ 4,000,000 KRW/month. ^5^ METs-hours/day. ^6^ kg/$m^{2}$. ^7^ Regular supplementation use (%). ^8^ kcal/day. ^9^ %E (percentage of energy from nutrients).

**Table 5:** Relative risks (RR) and 95% confidence intervals (95% CI) of hyper-LDL cholesterolemia according to tertiles (T) of the four FACP scores by sex ^1^.

	Tertile of Pattern Score			*p* for Trend	*p* for Interaction ^3^
	T1	T2	T3		
**Long-chain FA pattern**					
Total (*n* = 6542)					
Cases/person-years	511/19,930.1	520/19,886.7	471/20,382.7		
RR (95% CI) ^2^	1.00	1.09 (0.96–1.24)	1.05 (0.91–1.20)	0.651	0.791
Men (*n* = 3111)					
Cases/person-years	111/7137.0	183/10,297.3	205/12,463.6		
RR (95% CI)	1.00	1.12 (0.88–1.42)	1.04 (0.82–1.33)	0.975	
Women (*n* = 3431)					
Cases/person-years	400/12,793.1	337/9589.4	266/7919.0		
RR (95% CI)	1.00	1.09 (0.94–1.27)	1.06 (0.90–1.25)	0.530	
**Short and medium-chain SFA pattern**					
Total (*n* = 6542)					
Cases/person-years	470/20,325.2	449/20,380.6	583/19,493.6		
RR (95% CI)	1.00	0.92 (0.80–1.05)	1.17 (1.03–1.32)	0.004	0.042
Men (*n* = 3111)					
Cases/person-years	139/9889.8	1642/10,953.6	198/9054.5		
RR (95% CI)	1.00	0.93 (0.74–1.17)	1.34 (1.07–1.69)	0.003	
Women (*n* = 3431)					
Cases/person-years	331/10,435.4	287/9427.0	385/10,439.1		
RR (95% CI)	1.00	0.91 (0.77–1.07)	1.08 (0.92–1.26)	0.196	
**n-3 PUFA pattern**					
Total (*n* = 6542)					
Cases/person-years	487/20,404.1	485/20,338.6	530/19,456.8		
RR (95% CI)	1.00	0.90 (0.79–1.02)	0.96 (0.84–1.10)	0.809	0.071
Men (*n* = 3111)					
Cases/person-years	156/10,679.1	167/9938.3	176/9280.5		
RR (95% CI)	1.00	1.06 (0.85–1.33)	1.09 (0.87–1.37)	0.485	
Women (*n* = 3431)					
Cases/person-years	331/9725.0	318/10,400.3	354/10,176.3		
RR (95% CI)	1.00	0.83 (0.71–0.98)	0.91 (0.77–1.06)	0.427	
**Long-chain SFA pattern**					
Total (*n* = 6542)					
Cases/person-years	514/20,036.2	517/19,920.6	471/20,242.6		
RR (95% CI)	1.00	0.94 (0.83–1.06)	0.82 (0.72–0.94)	0.004	0.070
Men (*n* = 3111)					
Cases/person-years	165/10,022.8	187/9966.9	147/9908.2		
RR (95% CI)	1.00	1.01 (0.81–1.25)	0.73 (0.58–0.93)	0.007	
Women (*n* = 3431)					
Cases/person-years	349/10,013.4	330/9953.7	324/10,334.4		
RR (95% CI)	1.00	0.90 (0.77–1.05)	0.86 (0.73–1.01)	0.064	

^1^ RR and 95% CIs were obtained from Cox proportional hazard analysis. ^2^ Adjusted for age (5-year interval), sex, residence (rural or urban), smoking (never, past, or current), alcohol consumption (never, past, or current), supplementation use (yes or no), physical activity (METs-hours/day, quintiles), household income level (< 1,000,000, 1-<2,000,000, 2-<4,000,000, or ≥ 4,000,000 KRW per month), occupation (office worker, non-office worker, or house workers and others), BMI (< 18.5, 18.5-<23, 23-<25, or ≥ 25 kg/m^2^), and energy intake (kcal/day, quintiles). All sex-stratified models adjusted for the same variables, except for sex. ^3^ Test for interaction between sex and FACP.
